# Summarized datasheet for multi-omics response of three *Exaiptasia* strains to heat stress: a new way to process omics data

**DOI:** 10.1186/s13104-018-4018-x

**Published:** 2018-12-18

**Authors:** Maha J. Cziesielski, Yi Jin Liew, Manuel Aranda

**Affiliations:** 1 Red Sea Research Center, King Abdullah University of Science and Technology, Thuwal, Saudi Arabia; 2 Present Address: CSIRO Health & Biosecurity, North Ryde, NSW Australia

**Keywords:** *Exaiptasia*, Transcriptomics, Proteomics, Model organism, Biomarkers, Coral bleaching, Thermotolerance, Climate change, Coral reefs

## Abstract

**Objectives:**

Corals, the building blocks of reef ecosystems, have been severely threatened by climate change. Coral bleaching, the loss of the coral’s endosymbiotic algae, occurs as a consequence of increasing ocean temperature. To understand mechanisms of stress tolerance in symbiotic cnidarians, the sea anemone *Exaiptasia pallida* from different regions was heat stressed. The three strains originated from the Red Sea, Hawaii and North Carolina, each with different temperature profiles, enabling a comparative study of local adaptation strategies.

**Data description:**

Whole transcriptome and proteome data were collected from all anemones at control and stress condition. As part of the analysis of this large, multi-omic data, we wrote a script that creates a tabular datasheet that summarized the transcriptomic and proteomic changes for every gene. It facilitates the search of individual genes, or a group of genes, their up- or downregulation during stress and whether this change in expression was statistically significant. Furthermore, it enables examining if changes in RNA correspond to those in proteins. The datasheet can be used for future comparisons, as well as search and development of biomarkers.

## Objectives

Corals live in a symbiotic relationship with the algae Symbiodiniacea, which lives inside their tissue and provides corals with the majority of their energy demand. However, this relationship is fragile; particularly temperature stress can lead to the breakdown of this relationship, known as coral bleaching. Interestingly, a range of temperature tolerances can be found between and within species individuals, leading to some individuals being more susceptible to temperature increase than others. Particularly the habitat from which a coral originates can have an impact on its stress tolerance [1].

To understand what cellular mechanisms drive thermotolerance, how different genotypes have adapted to temperature and whether origin influences the stress response of symbiotic cnidarians, we conducted full transcriptome and proteome analysis of the coral-symbiosis model organisms the anemone *Exaiptasia*. Comprehensive analysis of the data and experimental details are described in Cziesielski et al. [2].

We created a datasheet that summarized all of our gene expression response on both transcriptomic and proteomic level. The spreadsheet eases data discovery, discern common patterns as well as differences in thermotolerance, thus aiding in hypothesis generation. While the raw data is freely accessible, it is far easier to access information summarized in this datasheet, especially for inter-study response comparisons, validation and biomarkers development. Through simply filtering columns for content, anyone can obtain entire transcriptome and proteome responses in a simple, yet informative, format. By making this datasheet available, we hope to contribute to facilitating collaborative progress in coral research, specifically regarding *Exaiptasia*, for researchers and educators alike.

We realized that this data format could be a useful tool to anyone working on large-omic datasets, as it condenses an extensive amount of sequencing information into an easy to use spreadsheet. In hopes of facilitating—omics data analysis across biological disciplines, we also provide the script used to generate the spreadsheet.

## Data description

Anemones originating from thermally different environments [North Carolina (CC7), Hawaii (H2) and the Red Sea (RS)] were maintained for over a year at control conditions (25 °C). For thermal stress, population subsets were gradually taken up to 32° and kept there for 24 h. Transcriptomes and proteomes were sequenced [3] and analyzed for stress response changes, as per Cziesielski et al. [2].

This summary datasheet contains *Exaiptasia* gene ID, gene annotations, statistical significance of expression changes and direction for each gene on transcript and protein level. To ease discovery, labels were used in the summary table instead of raw p-values. “Up” and “down” refer to the relative expression of the transcript/protein at 32 °C relative to the control condition (25 °C); the prefix “diff_” signifies differential expression, and added if the (multiple-testing corrected) p-value of the respective analyses were below 0.05. If the transcript or protein was not detected, no information was provided. Users can search and filter the data using any of the above categories in order to gather information about *Exaiptasia* genotype specific heat stress response on a multi-omics level (Table 1).

**Table 1:** Information on data files

Label	Name of data file	File types	Data repository identifier (DOI or accession number)
Data file 1	Summary datasheet of *Exaiptasia* heat stress response	MS Excel file (.xlsx)	10.5281/zenodo.1469124
Data file 2	Code and raw data used to produce summary datasheet	gzip-compressed tarball (.tar.gz)	10.5281/zenodo.1469124

Furthermore, we provide the code used to generate this summary sheet, with the hope that future studies will find value in creating summary sheets as presented here [4]. The script, implemented in Python 3, first reads in raw transcriptomic results (the comma-separated *.csv files in Data file 2) and raw proteomic results (the tab-separated “prot.fold_changes.tsv” in Data file 2). We noticed that quite a number of *Exaiptasia* gene models were duplicated—while this is biologically feasible, these are most likely a result of assembly artefacts. The inclusion of duplicate gene models, which would have identical functional annotations, could potentially bias downstream functional enrichment analyses. To remove this bias, our script reads in a set of whitelisted gene IDs generated in Cziesielski et al. [2], and removes genes outside this list. The custom script presented here is written to integrate two sets of—omics data.

From a technical point, the in-depth insight into transcriptome and proteome allows investigation into previously suggested biomarkers as well as evaluating new candidates. Many factors need to be kept in consideration and what works for one strain may not necessarily be the correct indicator in another, a factor rarely addressed in biomarker development [5]. Besides transcriptome–proteome interactions, developing and validating biomarkers need to consider that gene homologs respond differently to stress within and across genotypes. This can be observed using the datasheet, for example: glutathione peroxidase, commonly used as a biomarker in heat stress, has at least two homologs that significantly respond in all strains. However, both are significantly regulated in opposite directions (AIPGENE513, AIPGENE5657). Additionally, a gene that responds strongly in one genotype may not have a significant response in others. These limitations can inhibit the accuracy of data interpretation. By considering homolog and genotype response, the datasheet provides a source to make more informed decisions in biomarker usage.

## Limitations

This datasheet was made as a tool in order to utilize previously published data. As such, there are no major limitations. However, it should be kept under consideration that sequencing depth of the proteome is less than that of the transcriptome. While technology and analytical tools are quickly progressing, proteomic tools still do not keep up with sequencing efficiency of transcriptomics [6]. Sequencing depth is critical for correlation studies and comprehensive analysis of the cell. Low proteome coverage is often a result of detecting only abundant proteins and peptides, while low abundant proteins are not detected [7]. Furthermore, proteome changes are naturally time-dependent, and in light of protein misfolding due to heat stress likely further delayed [8], we cannot exclude time-lag as a potential factor for the absence in significant fold changes. Thus, we were unfortunately only able to sequence 12% of the proteome of *Exaiptasia* and could not find any significant differences in protein abundances in response to heat stress.

## Abbreviations

CC7: Anemones originating from North Carolina
H2: Anemones originating from Hawaii
RS: Anemones originating from the Red Sea
